# Attenuated contact heat-evoked potentials associated with sensory and social-emotional symptoms in individuals with autism spectrum disorder

**DOI:** 10.1038/srep36887

**Published:** 2017-01-31

**Authors:** Yi-Ling Chien, Shao-Wei Wu, Chih-Pang Chu, Sung-Tsang Hsieh, Chi-Chao Chao, Susan Shur-Fen Gau

**Affiliations:** 1 Department of Psychiatry, National Taiwan University Hospital and College of Medicine, Taipei, Taiwan; 2 Graduate Institute of Clinical Medicine, College of Medicine, National Taiwan University, Taipei, Taiwan; 3 Department of Neurology, National Taiwan University Hospital and College of Medicine, Taipei, Taiwan; 4 Department of Psychosomatic Medicine, Taipei City Psychiatric Center, Taipei City Hospital, Taipei, Taiwan; 5 Graduate Institute of Brain and Mind Sciences, College of Medicine, National Taiwan University, Taipei, Taiwan; 6 Department of Anatomy and Cell Biology, College of Medicine, National Taiwan University, Taipei, Taiwan

## Abstract

Sensory disturbance is associated with socio-emotional problems in individuals with autism spectrum disorder (ASD). Most studies assess sensory symptoms by self-reports, which are largely limited by the language ability and self-awareness of the individuals. This study aims to investigate sensory disturbance by contact heat-evoked potentials (CHEP) in ASD individuals, and to examine the clinical correlates of CHEP parameters. We compared the CHEP parameters and reported pain between 31 ASD individuals (aged 20.5 ± 5.2 years) and and 22 typically-developing controls (TD, aged 21.4 ± 2.6), and correlated the CHEP parameters with self-reported sensory symptoms and attention/socio-emotional symptoms. We found that ASD individuals showed smaller P2-wave amplitudes than TD, even though they reported a similar level of pain. In TD individuals, a smaller P2-wave amplitude was related to higher scores on ‘low registration,’ ‘attention to detail,’ and ‘attention switching difficulties.’ In ASD individuals, longer N2-wave latency was related to higher scores on ‘sensory sensitivity’ and socio-emotional problems; while higher reported pain was associated with higher scores on ‘low registration,’ overall autistic severity, and longer N2-wave latency. Our findings of attenuated CHEP response in ASD, which was associated with sensory symptoms and socio-emotional problems, suggest a potential role for CHEP in studying sensory disturbances in ASD.

Autism spectrum disorder (ASD) is characterized by two essential features, “social communication deficits” and “restricted, repetitive patterns of behavior/interests/activities”, with hyper- or hypo-reactivity to sensory input as one of the key symptoms^1^. Based on parental reports^2^ and self-reports^3^, sensory disturbances in ASD cover various domains of sensation, particularly tactile, under-responsiveness, and auditory filtering. Some experimental studies investigated sensory response by introducing vibration, heat, or cold stimuli, and demonstrated increased sensitivity to high-frequency vibration^4^ and thermal pain^5^ in ASD adults. Most of these studies relied on the self-reports of the intensity of sensation and/or observations of facial expression^6^. However, patients themselves may not appropriately report their sensory problems due to impairment in verbal communication^7^. A lack of objective sensory measures impedes our understanding of sensory processing aberrations in ASD.

Pain perception, a specific type of sensorium, is less appreciated in ASD based on anecdotal reports and clinical observation^7^; moreover, pain may not get recognized^8^ and treated in ASD^9^. Despite a lack of systematic studies on pain sensitivity and reactivity in ASD, the presence of pain insensitivity has been included in the DSM-5^10^ as an associated feature of “apparent indifference to pain/temperature,” and in the DSM-IV and DSM-IV-TR as “a high threshold for pain^11^.” The belief that children with ASD are insensitive to pain^8^ may bias observers’ judgments of the presence and severity of pain^6^. Such an assumption gained support from a recent review^12^ that youths with ASD do not necessarily have decreased sensitivity to pain because 5 in 10 experiments showed greater pain sensitivity in ASD^5^,^13^,^14^. As subjective measures may partly explain the controversial findings, an objective tool reflecting pain response without being influenced by language ability and culture is needed.

Contact heat-evoked potentials (CHEP) are a non-invasive neurophysiological recording of electrical activities in response to thermal stimuli through Aδ or C fibers^15^,^16^. The responses mediated by Aδ fibers can be consistently recorded at the vertex^17^, and have been applied in assessing the thermal pain pathway in diseases of the central^18^ or peripheral nervous system^19^. The CHEP methodology is well established^20^. Evidence has shown that attention towards stimuli might influence pain perception, e.g., refs 21 and 22. However, how the CHEP parameters correlate with sensory symptoms and attention characteristics in daily life is largely unknown. The clinical meanings of CHEP parameters in normal population are far from clear.

The interplay between sensory and emotional symptoms has been emphasized^23^. Sensory disturbance in ASD was highly associated with emotional dysregulation (e.g., more severe anxiety^24^), resulting in remarkable sufferings in daily life^25^. On the contrary, emotion may also influence pain sensitivity^26^ by dampening or amplifying signals, e.g., ref. 27, serving as a significant modulating factor for pain perception. Evidence from functional MRI shows that emotional empathy positively correlates with brain activation in areas involved in an embodiment of pain in ASD^28^, which sheds light on the interrelationship between pain perception and social emotion. In daily life, individuals with ASD have difficulty in handling social situations; that may be related to multiple sensory inputs because a social context is usually changeable, unpredictable and often emotionally arousing^22^. New evidence also suggests that brain regions that process sensory input were frequently engaged during affective experiences driven by sensory inputs^29^. In this regard, whether individuals with socio-emotional problems also demonstrate abnormal CHEP responses is of clinical significance.

This study aims to investigate the sensory disturbances in ASD and to examine the sensory and attention correlates of CHEP and the perceived pain by combining objective (CHEP) and subjective measures. We also test whether the CHEP responses are associated with socio-emotional problems in ASD. We hypothesize that individuals with ASD, compared to typically-developing controls (TD) would show different responses on CHEP, which would correlate with self-reported daily sensory symptoms and attention characteristics. Meanwhile, we assumed that the neural correlate (CHEP response) and clinical correlates of subjective pain severity, including sensory symptoms, attention characteristics and autistic symptoms, may be associated with the severity of socio-emotional problems in individuals with this disorder.

## Results

### Autistic and Sensory Symptoms

Compared to TD participants, ASD participants showed significantly higher scores for stereotyped behaviors and socio-emotional problems (on the questionnaire ‘Social Responsiveness Scale’, SRS), attention to detail and attention switching difficulty (on ‘Autism Spectrum Questionnaire’, AQ), and higher total scores on SRS and AQ (Table 1). They also reported higher symptom severity on Low Registration, Sensory Sensitivity, and Sensation Avoiding (on ‘Sensory Profile,’ SP) than TD participants (Table 1). However, ASD participants reported lower Sensation Seeking scores (on SP) than TD controls (Table 1).

### CHEP and Verbal Rating Scale (VRS)

**Figure 1:**
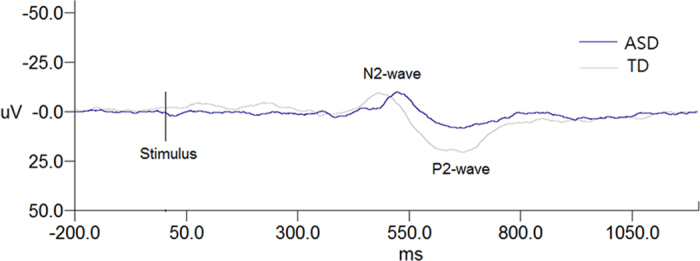
Grand average of CHEP waves of the group with autism spectrum disorder (ASD: dark line) and the group with typically-developing participant (TD: light gray line). The N2-wave and P2-wave were marked. The ASD participant showed a smaller P2-wave amplitude.

Figure 1 presents the grand average of CHEP parameters for the ASD and TD groups. In general, participants with ASD had smaller amplitudes of the P2-wave of CHEP than TD participants. We further compared the latencies and amplitude of N2- and P2-waves between the two groups and found a smaller P2-wave amplitude in ASD than TD (Table 2). The significance remained after controlling for sex, age, and full-scale IQ (Table 2). P2-wave latency and N2-wave parameters were not significantly different between the two groups when controlling for sex and age (Table 2). There was no group difference in reported pain intensity on the VRS, either (Table 2).

### Sensory and attention correlates of P2-wave amplitude

**Figure 2:**
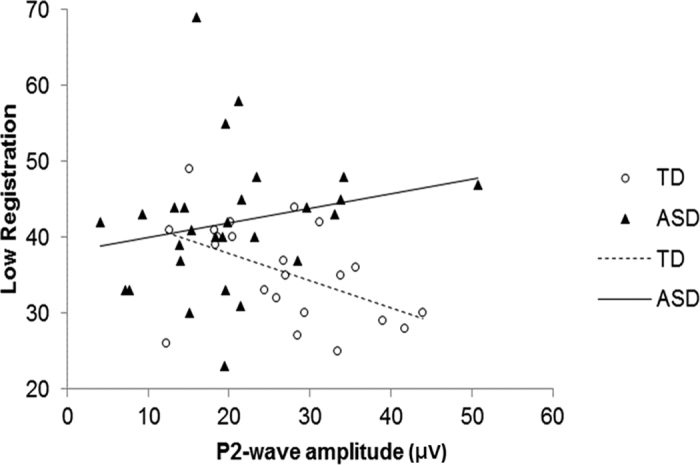
Correlations between P2-wave amplitude and Low Registration subscores in individuals with autism spectrum disorders (ASD) and typically-developing controls (TD). Diagnosis group had a main effect (β = −40.0 ± 13.6, F = 8.59, p = 0.005) with a significant interaction between group and Low Registration (β = 0.91 ± 0.36, F = 6.45, p = 0.015). The results were similar when the seemly outlier of an ASD participant with a very high amplitude was deleted [Diagnosis group main effect (F = 10.04, p = 0.003), interaction term (F = 7.03, p = 0.010)].

We examined the sensory correlates of P2-wave amplitude among the TD group in order to find the sensory characteristics that may influence P2-wave amplitude in normal population. First, to identify the most important sensory correlate, four SP subscores were included in the stepwise model selection. A lower P2-wave amplitude was associated with a higher score on Low Registration (β = −0.68 ± 0.27, F = 6.50, p = 0.019). When controlling for the confounding effects, the group difference between ASD and TD regarding the Low Registration subscores and the group × Low Registration interaction remained significant (Fig. 2). In contrast, for ASD participants, a lower P2-wave amplitude showed a trend to correlate with lower scores on Low Registration and Sensory Sensitivity (Supplementary Table 2).

For the attention correlates of the P2-wave amplitude, we found negative correlations between lower P2-wave amplitudes and higher scores in attention to detail (*r*_*s*_ = −0.49, p = 0.022) and attention switching difficulty (*r*_*s*_ = −0.53, p = 0.012) in the TD group but no such correlations in the ASD group. If we controlled either ‘attention to detail’ or ‘attention switching difficulty’, the group difference of P2-wave amplitudes between ASD and TD disappeared (p = 0.150 or 0.615).

### Clinical correlates of reported pain intensity

**Figure 3:**
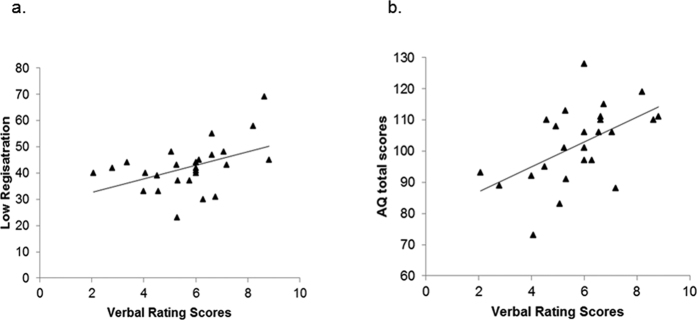
Relationship between reported pain intensity on the Verbal Rating Scale and (**a**) Low Registration subscores (**b**) Autism Spectrum Quotient (AQ) total scores in individuals with autism spectrum disorders. Reported pain intensity was positively correlated with Low Registration subscores (*r*_*s*_ = 0.46, p = 0.015) and AQ total scores (*r*_*s*_ = 0.52, p = 0.006).

To analyze the clinical correlates of reported pain in the ASD group, four SP subscores, attention characteristics, and the overall severity of autistic symptoms (AQ total scores) were included in the stepwise selection model. Finally, Low Registration (β = 0.07 ± 0.03, F = 6.70, p = 0.017) and AQ total scores (β = 0.06 ± 0.02, F = 8.60, p = 0.008) remained in the model to be positively associated with a higher reported pain intensity in ASD. However, the reported pain intensity was associated with higher scores on both symptoms only in ASD (Fig. 3) but not in TD, resulting in symptom × group interactions (AQ total × group: β = 0.10 ± 0.04, F = 5.74, p = 0.021; Low Registration × group: β = 0.16 ± 0.06, F = 6.84, p = 0.012).

### CHEP correlate of reported pain intensity

To examine the CHEP correlate of reported pain in ASD, we found that among the four CHEP parameters, N2-wave latency was the only parameter to be significantly associated with reported pain intensity (β = −0.02 ± 0.00, F = 7.01, p = 0.014) that shorter N2-wave latency was related to higher reported pain severity. To explore the sensory correlate of N2-wave latency in ASD, Spearman’s correlation test showed that shorter N2-wave latency was correlated with higher scores on Sensory Sensitivity (*r*_*s*_ = −0.50, p = 0.011, Supplementary Table 2).

### N2-wave latency vs. socio-emotional problems

**Figure 4:**
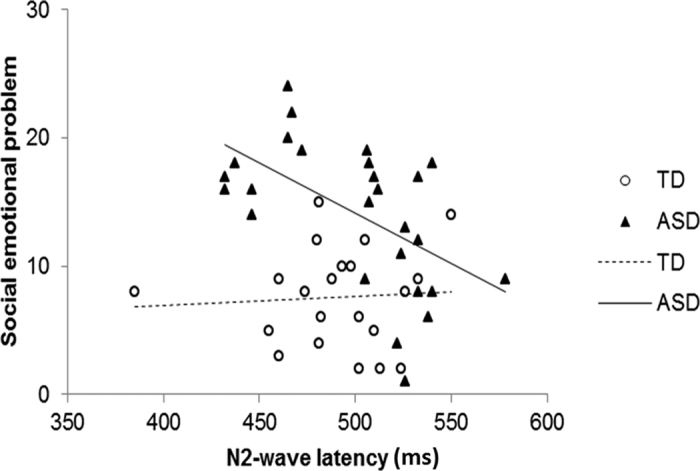
Correlations between N2-wave latency and social emotion problem subscores in individuals with autism spectrum disorders (ASD) and typically-developing controls (TD). Diagnosis group had a main effect on socio-emotional problems (β = 49.3 ± 18.0, F = 7.48, p = 0.009), and N2-wave latency showed a nominal effect (β = 0.007 ± 0.029, F = 3.91, p = 0.054) with significant group × N2-wave latency interaction (β = −0.086 ± 0.036, F = 5.54, p = 0.023). The results were mildly different when the outlier of a TD participant with a very short latency was deleted [diagnosis main effect (F = 5.57, p = 0.023), N2-wave latency main effect (F = 1.77, p = 0.191), and interaction term (F = 4.28, p = 0.045)]. When ASD and TD were separated, the N2-wave latency was still associated with socio-emotional problems significantly in ASD group (t = −3.28, p = 0.0032) but not in TD group (t = 0.27, p = 0.792).

Since N2-wave latency was the only CHEP parameter to associate with reported pain and was negatively correlated with Sensory Sensitivity in ASD, we tested the relationship between N2-wave latency and socio-emotional problems. We found that shorter N2-wave latency was correlated with higher socio-emotional problems (*r*_*s*_ = −0.54, p = 0.005) in ASD. When we combined the ASD and TD groups together, N2-wave latency was still significantly associated with socio-emotional problems when the ASD diagnosis was controlled (F = 6.39, p = 0.015). When testing for diagnosis × N2-wave latency interaction, the presence of ASD diagnosis and N2-wave latency had nominal main effects on socio-emotional problems with a statistically significant group × N2-wave latency interaction (Fig. 4). This model explained 47% of the variance in socio-emotional problems (R^2^ = 0.47, F = 12.53, p < 0.0001). Considering those socio-emotional problems were associated with sensory symptoms (Supplementary Table 3), we adjusted the sensory symptoms and tested the relationship in the ASD group. We found that shorter N2-wave latency was still significantly associated with higher socio-emotional problems in ASD even when all the four sensory symptoms were adjusted in the regression model (β = −0.09 ± 0.03, t = −3.50, p = 0.0024), but was no longer significant in TD (p = 0.482).

## Discussion

As the first study to explore the clinical significance of thermal pain measured by the CHEP in individuals with ASD, we found that compared to TD individuals, ASD individuals significantly had a smaller P2-wave amplitude, which may relate to self-reported sensory symptoms and attention characteristics in daily life. Individuals with ASD did not report a different level of pain on CHEP, but the pain severity was positively correlated with overall autistic symptoms. The N2-wave latency, which was the only CHEP parameter to correlate with reported pain severity in ASD, was associated with self-reported sensory sensitivity and socio-emotional problems in ASD. Our findings provide new evidence to interpret CHEP parameters regarding daily sensory symptoms, suggesting a potential role for CHEP in studying sensory disturbances in ASD.

Our novel finding of a decreased P2-wave amplitude on CHEP in ASD individuals implies an attenuated physiological response to thermal stimulation in ASD. Different CHEP responses in ASD could be caused by dysregulation in either the central^18^ or peripheral^19^ thermal nociceptive pathways. The N2- and P2-wave of CHEP are supposed to originate mainly from the cingulate gyrus^17^, which receives spinothalamic inputs via the thalamic nucleus and mediates the affective component of pain^17^,^27^. A recent fMRI study located a neurologic signature of CHEP, including several regions such as the secondary somatosensory cortex and the anterior cingulate cortex (ACC)^30^. P2-wave component primarily reflects the neuronal activity of ACC^17^ that works to integrate sensory information and direct attention to the stimuli^31^. The ACC, an area supposed to be activated by both innocuous and noxious heat stimulation^32^, is also implicated in ASD based on evidence of abnormal activation^33^ and connectivity^34^. Dysregulation of ACC may potentially be one of the mechanisms that explain the reduced P2-wave amplitude in ASD, reflecting on a possibility that inadequate activation to properly integrate the sensory information results in physiological hyposensitivity to pain. Future functional MRI study to examine the brain activation during CHEP may help to elucidate the underlying mechanism. Furthermore, ACC may correspond to the top-down attentional modulation of sensory processing^35^. Some researchers have argued that the reduced P2-wave amplitude may also reflect inattention from stimulation, voluntarily or automatically^21^. In our study, all the participants were instructed to pay attention to the stimuli and reported the perceived pain one by one. The effect on attention was hard to evaluate unless a distraction test was employed. Moreover, other factors, including skin innervation, thermal thresholds, and sensory nerve conduction, may influence CHEP responses^20^. Nevertheless, our finding suggests that CHEP might be a physiological indicator of sensory disturbance for ASD in adolescence and early adulthood. To validate CHEP as a sensory biomarker, our next step will be to investigate CHEP responses to sensory stimulation besides thermal pain, and to apply CHEP to other developmental conditions with sensory processing problems to examine its specificity to ASD will be our next step. Also, a longitudinal study to examine the changes of CHEP responses with age may help to clarify its stability.

As the first study to examine the behavioral correlates of CHEP measures in normal participants, our findings of smaller P2-wave amplitudes associated with low registration, attention to detail, and worse attention switching measured by questionnaires implied a tendency to local processing and high-threshold sensory responses. In this sense, the reduced P2-wave amplitudes of ASD individuals in our sample could be related to their under-responsiveness and attention characteristics, in that they were at the extreme end on the subscale of Low Registration, attention to detail, and attention switching difficulty compared to normal participants. Similarly, a recent study demonstrated that decreased thermal sensitivity in ASD might be associated with cognitive impairments relating to attention reorienting problems^36^, echoing a previous report using cluster analysis to display the sensory correlates of overfocused attention^22^. It is still unclear whether the reduced P2-wave amplitude we observed is due to autistic neuropathology itself or the habitual allocation of attention. Our findings showed that the significance of the group difference in the P2-wave amplitude disappeared when attention characteristics were controlled, suggesting that the reduced P2-wave amplitude may be modulated by the attention characteristics inherent to ASD. Switching attention involves appropriate functioning of dorsolateral prefrontal cortex, e.g., ref. 37, while latest studies suggest that ACC may coactivate/synchronize with prefrontal cortex for top-down attentional control in conflict stimuli, attention shifts, or multi-sensory integration^38^,^39^,^40^,^41^,^42^,^43^. As P2-wave primarily reflects the activation of ACC, our findings provide new evidence to support the association between ACC activity and pain-triggered attention that has been demonstrated by the dipole model of CHEP topography in normal participants^16^. Despite unclear mechanism, the role of ACC as attention modulation in mediating CHEP responses is intriguing and worthy of further research.

Our findings of deviated CHEP responses without different severities of reported pain in ASD are consistent with a recent study showing greater heart rate response and elevated beta-endorphin upon venipuncture without behavioral pain reactivity, suggesting a different mode of pain expression in ASD^7^. Meanwhile, the reported pain intensity in ASD was associated with the Low Registration subscores, suggesting that patients who tended to ignore stimuli in daily life reported higher pain scores during CHEP. Perhaps they are not as lacking in feelings as their behaviors appear to indicate, but selectively “escape” from stimuli to avoid strong physiological reactions. This finding was also in keeping with a previous hypothesis that the unusual sensory responsiveness may be related to stimulus over-selectivity, in which individuals with ASD tend to respond only to a limited amount of relevant sensory information^44^. Furthermore, the finding of the association between reported pain intensity and AQ total scores in ASD suggests that perceived pain increased with more severe overall autistic symptoms. These findings were in contrast to the belief that ASD individuals were insensitive to pain^8^. Although they did not report higher pain severity during CHEP, the perceived pain may still be higher in those with more severe overall autistic symptoms and under-responsiveness. Combined with the reduced P2-wave amplitude in ASD, the construct of hyper- or hypo-sensitivity might be over-simplified to understand this complicated yet strikingly heterogeneous phenomenon. Likewise, several studies that administered the Sensory Profile in ASD also demonstrate a combination of low registration (or under-responsiveness) and sensory sensitivity (or hyper-responsiveness) across the sensory modalities^45^,^46^,^47^,^48^.

The association between N2-wave latency and reported pain intensity in ASD implied that the earlier physiological response might correlate with greater perceived pain, consistent with our previous findings in normal population^7^. The findings that N2-wave latency correlated with sensory sensitivity and higher socio-emotional problems in ASD suggest its potential role as a salient physiological indicator for a sensory disturbance in ASD. In previous laser-evoked potential studies, the N2-wave component originated mainly from the ACC^49^, and may also reflect neuronal activity in the primary or secondary sensory cortex as well as the dorsal posterior insula^50^,^51^. The activation of ACC may play an integrative role, directing attention to the stimuli and preparing a motor reaction^31^, while sensory cortex activation was specifically involved in the recognition of the noxious nature of stimuli^52^. Patients with earlier N2-wave reported more severe thermal pain and daily sensory symptoms, and also suffered from more socio-emotional problems, suggesting a mechanism involving not only sensory integration (ACC), but also the recognition of noxious stimuli (sensory cortex) that may likely underlie these associations. Our findings might provide physiological evidence to explain the association between sensory sensitivity and socio-emotional problems observed in ASD^28^. Further neuroimaging studies to investigate structural or functional connectivity between brain regions for emotion regulation and the sensory system may help to elucidate their interrelationship. Moreover, earlier N2-wave was associated with more severe socio-emotional problems when the diagnosis was controlled and, for the ASD group when sensory symptoms were adjusted, that highlights a specific relationship between N2-wave latency and socio-emotional problems independent of the diagnosis and the severity of sensory symptoms. Sensory characteristics seem to mediate the relationship between N2-wave latency and socio-emotional problems in normal subjects, as expected, but not in ASD individuals, a novel finding that is worth validation. Our results set up a starting point to study the role of CHEP in socio-emotional processing.

This study has several limitations. First, attention towards stimuli was not controlled in the experiments. Instead, we assessed attention by self-reports, showing a relationship between the P2-wave amplitude and attention towards daily stimuli. Future studies may focus on the contralateral temporal N1 component, which is less sensitive to attention, or considers a distraction test during CHEP to control for attention. Second, there was compromised generalizability due to the small sample with male-predominance. Hence, we analyzed the data using non-parametric methods to generate a more conservative report. Third, sex differences were noted in the ASD brain, e.g., ref. 53. However, a small number of female participants prevented us from conducting subgroup analyses or drawing a conclusion regarding a gender effect in the CHEP response. Therefore, we controlled for the sex in the statistical analysis to avoid any possible confounding effects. Third, we assessed socio-emotional problems by self-reports to capture the subjective experience in daily life and the first author assisted to ensure that participants can understand the questions clearly. Other sources of information (e.g. parent-report or clinician-rated) or applying social emotion tasks may provide objective observations on socio-emotional problems and may be considered in the future study. Last, other factors, such as skin innervation, thermal thresholds, and sensory nerve conduction, may influence CHEP responses^20^. Further studies will be required to dissect the structural substrates.

In summary, the findings of this novel report imply a smaller P2-wave amplitude on CHEP in individuals with ASD, which correlate with daily sensory symptoms, as an important physiological indicator for sensory disturbances in ASD. The reported thermal pain of individuals with ASD, though not different from that of TD controls, was influenced by the overall autistic symptom severity, and was related to socio-emotional problems. Clinical pain assessment relying solely on self-reports could be misleading; a comprehensive evaluation including daily sensory symptoms, the severity of autistic symptoms, and physiological measurement is important in assessing pain response in individuals with ASD. Moreover, the unique relationship of an earlier N2-wave in ASD with sensory sensitivity and reported thermal pain suggests that the brain function of sensory regulation may modulate the clinical presentation of ASD. Our findings also provide the first explorative evidence of behavioral correlates of CHEP in normal participants, showing P2-wave parameters as the potential indicators of attention to external stimuli. These findings warrant validation and further research on the physiological implications.

## Methods

### Participants and Procedure

We recruited 31 participants diagnosed with ASD (29 males, aged 20.5 ± 5.2 years) according to DSM-IV criteria and 22 TD controls (20 males, 21.4 ± 2.6 years). The diagnosis was confirmed by the first author and the corresponding author (SSG), board-certificated child psychiatrists, using the DSM-5 criteria and the Autism Diagnostic Interview-Revised (ADI-R)^54^,^55^ (Supplementary Table 1). The age and sex distribution and the years of education of the participants did not differ between the two groups (Table 1). Participants with ASD had a significantly lower IQ than the TD had on the Wechsler Adult Intelligence Scale-IV (Table 1).

The study was implemented after the approval by the Research Ethics Committee of National Taiwan University Hospital (NTUH) (ID: 200809066 R). The methods were carried out by the approved guidelines. Written informed consent was obtained from all the participants and their parents (if the participants were younger than 20 years old) after full explanation of the objectives and procedure of the study. Participants with ASD were recruited from the Department of Psychiatry of NTUH while TD participants were recruited through advertisements. Each TD participant was clinically assessed to exclude any possibility of ASD or other psychiatric disorders. All the participants received the CHEP assessment and completed the Chinese versions of the Sensory Profile (SP)^56^ for sensory symptoms and the Social Responsiveness Scale (SRS)^57^ and the Autism Symptom Questionnaire (AQ)^58^ for autistic symptoms by themselves in the hospital. All participants did not take any medications during the 24 hours before the CHEP procedure.

### Measures

The SP^56^ is a widely used self-report questionnaire measuring sensory/perception-related symptoms and behaviors. The SP consists of 60 items covering six sensory modalities: taste/smell, motion, visual, tactile, activity, and auditory—with 10 items for each sensory modality. Rating is based on a five-point Likert scale [almost never (5%), seldom (25%), sometimes (50%), frequently (75%), and always (100%)]. The scoring method is based on a 4-dimensional structure— i.e., Low Registration (15 items, e.g., do not notice when being greeted/touched), Sensation Seeking (15 items, e.g., like to add pepper/chili in food), Sensory Sensitivity (15 items, e.g., dislike being touched), and Sensation Avoiding (15 items, e.g., escape from places with crowds). The psychometric properties of the Chinese-language version of the SP have been described elsewhere^59^; the Chinese-language SP has been used to assess sensory symptoms in adult populations in Taiwan.

The *SRS*^57^ is a 65-item rating scale that measures the severity of autistic symptoms. Items are rated by the subject on a four-point Likert scale from 0 (not true), 1 (sometimes true), 2 (often true), to 3 (almost always true). The Chinese-language SRS has demonstrated a satisfactory four-factor structure with high internal consistency (Cronbach’s alpha, 0.94–0.95), i.e., social communication, autistic mannerism, social awareness, and social emotion^60^. The Chinese SRS has been widely used to assess autistic traits in Taiwan. The subscale of social emotion was analyzed in this study to test its relationship with the CHEP correlate of reported pain. This subscore is a summation of 8 items; examples are item 1, “much more fidgety in social situation than when alone”, item 5, “doesn’t recognize when others take advantage”, item 46, “overly serious facial expressions”, item 60, “emotionally distant” and item 64, “too tense in social settings”.

The *AQ*^58^ is a self-report questionnaire developed to quantify autistic traits in general population. Each item is rated on a four-point scale with answer categories of “almost always true”, “often true”, “sometimes true” and “not true”. Every response is scored ‘1’ if “almost always true” or “often true” and ‘0’ if “sometimes true” or “not true”, leading to a total AQ score ranging from 0 to 50, in which the higher scores represent the autistic end of the continuum. The psychometric properties of the Chinese-language version of the AQ have been described elsewhere^58^. The AQ in Chinese-language has been used to measure autistic traits in adult populations in Taiwan. This study used the subscores of attention to detail, attention switching difficulty, and the total score to assess attention characteristics and overall autistic traits, respectively.

### CHEP paradigms

A contact heat-evoked potential stimulator (Medoc, Ltd., Ramat Yishai, Israel) was used to deliver heat stimulation^15^. The thermode with a circular contact area of 27 mm in diameter (572 mm^2^) comprised two layers of stimulators. The external layer consists of a heating thermofoil (Minco Products, Inc., Minneapolis, Minnesota), and is covered with a 25-μm layer of thermoconductive plastic (Kapton, thermal conductivity at 23 °C of 0.1 ± 0.35 W/m/K). Two thermocouples (electronic thermal sensors), embedded at 10 μm within, contacted the skin and provided an estimate of skin temperature^15^,^16^. The lower layer is a peltier element with two electronic thermal sensors and thermistors. The external thermofoil permitted a rapid heating rate, up to 70 °C/s, and the lower layer peltier allowed a fast cooling rate, up to 40 °C/s. Cooling began immediately after the thermode reached its target stimulus temperature.

CHEP was evoked by methods described previously^20^. In brief, participants lay on a bed, with their eyes closed, in a semi-dark room with the room temperature controlled to 25 °C. The stimulation site was in the right lateral leg 10 cm proximal to the lateral malleolus. To minimize the habituation effects of repetitive stimulation, the skin area of right lateral leg was divided into four adjacent non-overlapping districts and the thermode was moved clock-wise or counter-clockwise across these sites which were balanced between the participants. The baseline temperature of the thermode was set at 32 °C for all stimuli, and the heat pulse was delivered from the baseline to 51 °C. The inter-stimulus intervals were randomly set at 20 to 22 seconds. To avoid the startle due to the contact heat stimulus, we delivered 2~3 contact heat stimuli at the skin other than a right lateral leg to let the participants get used to the noxious stimulus before the formal recording. To ensure attention during the CHEP procedure, participants were asked to pay attention to the stimulation site throughout the study. The electroencephalogram (EEG) was continuously measured from 32 scalp electrodes that were mounted on an elastic cap according to the extended 10–20 system (32-channel Quik-Cap, *Neuroscan*, Charlotte, NC). The electrode impedance was kept less than five kΩ. Eye blinks and vertical eye movement were monitored by additional orbital electrodes around both eyes. Reference electrodes were applied bilaterally on earlobes. EEG data was registered using a Neuroscan SynAmps system (*Neuroscan*, Charlotte, NC). The sampling rate was 1000 Hz with a bandpass filter at 0~300 Hz. During the recording, participants were asked to verbally rate the intensity of pain perception 3 s after each stimulus. Pain intensity was reported using the Verbal Rating Scale (0–10), in which 0 = no sensation, 4 = pain threshold, and 10 = intolerable pain. At least 30 consecutive contact heat stimuli were applied to the recording of CHEP.

The offline processing of CHEP waveforms was based on Scan 4.5 software (NeuroScan, El Paso, Texas). The EEG signals in each channel were cut into epochs from 200 ms before the stimulus onset (0 ms) to 1200 ms after the stimulus onset. Each epoch was baseline corrected from − 200 to 0 ms and was filtered through a bandpass filter at 0.1~30 Hz. We inspected all epochs, and the epochs with major artifacts from eyeball movement, eye blink or muscle contraction were excluded. The analysis of CHEP waveforms was based on an average of the first 16 artifact-free epochs, and this number was determined based on the protocol of previous CHEP studies^15^,^19^,^61^,^62^. Because the early component (N1-P1) of CHEP is inconsistently recorded and has small amplitude^16^,^63^, the main and clinically useful component of CHEP signal is the widespread negative-positive complex (N2-P2) with maximum amplitude at the vertex as described previously^15^. The present report was based on data recorded from the Cz (vertex) electrode. We measured the latencies and amplitude of N2-wave and P2-wave automatically in the time window between 300 and 800 ms by the “peak detection” command in the Scan 4.5 software for further analysis. To define the background activities, we performed control test in each subject before the formal recording of CHEP. The recording of control test was the same as the formal CHEP recording described above except that the stimulator was applied to the skin without actual heat stimulation. The negative or positive background activity were defined as the mean negative or positive amplitude in the time window between 300 and 800 ms in the averaged tracing of the control test according to our protocol reported before^19^,^64^. When the amplitude of N2-wave or P2-wave was less than 2-fold of the negative or positive background activity, the N2-wave or P2-wave was considered to be absent, and the amplitude of N2-wave or P2-wave was defined as the negative or positive background activity.

### Data Analysis

We used SAS program 9.2 (SAS Institute Inc, Cary NC, USA) for statistical analysis. The SP, SRS, and AQ subscores, as well as the latency, amplitude, and mean VRS scores of the CHEP were expressed as mean ± SD. The CHEP parameters, the subscores and total scores of the ASD and TD groups were compared using the Wilcoxon rank sum test and the general linear model controlling for age, sex, and/or full-scale IQ. Spearman’s correlations (*r*_*s*_) between the CHEP parameters and SP and AQ attention subscores were computed for the ASD and TD groups, respectively, before multiple regression analyses. Model selections for P2-wave amplitude and VRS scores were performed by a stepwise selection with p < 0.10 entry and p < 0.05 staying in the model. To examine the behavioral correlates of VRS scores (AQ total and Low Registration) and CHEP correlate (N2-wave latency) for socio-emotional problems, main effects of the diagnosis group and the correlate, and their interaction term (group × correlate) were tested for each variable (i.e., VRS scores and socio-emotional problems). Besides, we also adjusted sensory subscores to see whether the N-wave latency was still associated with socio-emotional problems. Also, we further controlled attention characteristics when comparing P-wave amplitude to see whether attention characteristics can influence group difference. Statistical significance was set at p < 0.05. Bonferroni corrections were done in the correlation analyses of the four SP subscores (significance level p < 0.0125).

## Additional Information

**How to cite this article**: Chien, Y.-L. *et al*. Attenuated contact heat-evoked potentials associated with sensory and social-emotional symptoms in individuals with autism spectrum disorder. *Sci. Rep.* **7**, 36887; doi: 10.1038/srep36887 (2017).

**Publisher's note:** Springer Nature remains neutral with regard to jurisdictional claims in published maps and institutional affiliations.

## Supplementary Material

Supplementary Tables

## Figures and Tables

**Table 1:** Comparing the demographics, and autistic§ and sensory¶ symptom subscores between the autism spectrum disorder and typically-developing groups.

	ASD (N = 31)		TD (N = 22)		Statistics	*p*
	Mean or N	SD or (%)	Mean or N	SD or (%)		
Male (N, %)	29	(93.5%)	20	(90.9%)	0.13	0.720
Age	20.5	5.2	21.4	2.6	0.47	0.498
Full-scale IQ	101.4	19.1	114.1	10.2	2.49	0.013
Verbal IQ	104.1	18.6	114.5	9.7	2.07	0.039
Performance IQ	98.3	20.3	111.4	12.5	2.33	0.020
Social Responsiveness Scale						
Socio-emotional problem	14.6	5.5	7.4	3.9	−4.26	<0.0001
Autism Spectrum Quotient						
Attention to detail	11.4	2.3	9.6	1.7	−3.00	0.003
Attention switching	17.9	2.7	14.5	2.4	−3.91	<0.0001
Total scores	101.7	12.0	77.8	9.9	−5.15	<0.0001
Sensory Profile						
Low Registration	41.9	9.1	35.5	6.6	−2.76	0.006
Sensation Seeking	39.0	7.5	44.6	5.9	2.89	0.004
Sensory Sensitivity	44.6	10.9	37.8	7.7	−2.30	0.022
Sensation Avoiding	44.5	9.0	39.4	5.8	−2.27	0.023
Autism Diagnostic Interview-Revised						
Social reciprocity (severe)	17.5	7.4				
Communication: nonverbal (severe)	7.2	3.7				
Communication: verbal (severe)	13.5	5.1				
Restricted, repetitive, and stereotyped behaviors (severe)	6.5	2.8				
Social reciprocity (current)	9.6	4.3				
Restricted, repetitive, and stereotyped behaviors (current)	4.7	2.2				

**Note.** ASD: autism spectrum disorders, TD: typically-developing controls. The group comparison was tested by Wilcoxon rank sum test.

^§^Social Responsiveness Scale subscores and Autism Spectrum Quotient subscores. In Autism Diagnostic Interview-Revised, the abnormalities observed as the most severe conditions when the children were 4–5 years old passed the diagnosis cutoffs of 10, 8, 7, and 3, respectively, except for verbal communication.

^¶^Sensory Profile subscores.

**Table 2:** Comparison of contact heat-evoked potential parameters in the autism spectrum disorder and typically-developing groups.

	ASD (N = 31)		TD (N = 22)		Statistics§	*p*§	Adj.¶ F	Adj.¶ *p*	Adj.† F	Adj.† *p*
	Mean	SD	Mean	SD						
N2-wave latency	501.5	40.0	490.6	34.7	−1.23	0.220	0.86	0.358	0.18	0.673
N2-wave amplitude	−15.1	8.1	−13.4	8.4	1.31	0.190	0.11	0.743	0.79	0.379
P2-wave latency	675.6	51.2	651.5	35.6	−1.96	0.050	3.09	0.085	9.63	0.004
P2-wave amplitude	20.7	10.2	26.6	9.0	2.10	0.036	5.91	0.019	5.41	0.025
Verbal rating scale	5.6	1.6	5.5	1.8	−0.06	0.956	0.13	0.724	0.89	0.351

**Note.** ASD: autism spectrum disorders, TD: typically-developing controls.

^§^Non-parametric analysis by Wilcoxon rank sum test.

^¶^General linear model adjusted for sex and age.

^†^General linear model adjusted for sex, age, and full-scale IQ.
