# Supplementary material for: Attenuated contact heat-evoked potentials associated with sensory and social-emotional symptoms in individuals with autism spectrum disorder
Source: Sci Rep. 2017 Jan 31;7:36887. doi: 10.1038/srep36887 (PMC5282530; doi:10.1038/srep36887)
Supplement: Supplementary Tables [file srep36887-s1.doc]

**Title: Attenuated contact heat-evoked potentials associated with sensory and social emotional symptoms in individuals with autism spectrum disorder**

Author list: Yi-Ling Chien, MD, Shao-Wei Wu, MD, Chih-Pang Chu, MD, Sung-Tsang Hsieh, MD, PhD, Chi-Chao Chao, MD, PhD, Susan Shur-Fen Gau, MD, PhD

**Table 1. The subscores of Autism Diagnostic Interview-Revised (ADI-R) in the participants with autism spectrum disorders (ASD, N = 31)**

| **ADI-R subscores** | Mean | SD |
| --- | --- | --- |
| ***Most severe condition at 4-5 years old*** |  |  |
| A: Social reciprocity | 17.5 | 7.4 |
| B: Communication: nonverbal | 7.2 | 3.7 |
| Communication: verbal | 13.5 | 5.1 |
| C: Restricted, repetitive, and stereotyped behaviors | 6.5 | 2.8 |
| ***Current condition*** |  |  |
| A: Social reciprocity | 9.6 | 4.3 |
| C: Restricted, repetitive, and stereotyped behaviors | 4.7 | 2.2 |

**Note.** The abnormalities observed as the most severe conditions when the children were 4-5 years old passed the diagnosis cutoffs of 10, 8, 7, and 3, respectively, except for verbal communication. The current condition of B domains (communication) was not presented because the scoring algorithms were different according to developmental level, based on the coding manual, and was not suitable for average.

**Table 2. Spearman’s correlations (*r_s_* ) between contact heat-evoked potential parameters and sensory subscores in the autism spectrum disorder group**

|  | N2-wave latency | | P2-wave amplitude | |
| --- | --- | --- | --- | --- |
|  | *r_s_* | Uncorrected *p* | *r_s_* | Uncorrected *p* |
| Low Registration | -0.41 | 0.044 | 0.39 | 0.043 |
| Sensation Seeking | -0.26 | 0.217 | -0.01 | 0.959 |
| Sensory Sensitivity | -0.50 | 0.011* | 0.44 | 0.019 |
| Sensation Avoiding | -0.40 | 0.046 | 0.35 | 0.071 |

*Significant after Bonferroni correction (for the multiple tests on four sensory subscores, significant level 0.05/4, *p* < 0.0125).

**Supplementary Table 3. Spearman’s correlations (*r_s_* ) between socio-emotional problems and sensory characteristics in the whole sample**

|  | Socio-emotional problems | |
| --- | --- | --- |
|  | *r_s_* | *p* |
| Low Registration | 0.41 | 0.005 |
| Sensation Seeking | -0.10 | 0.505 |
| Sensory Sensitivity | 0.49 | 0.001 |
| Sensation Avoiding | 0.41 | 0.004 |
